# A Comparison of Long-Term Right Ventricular Functions in Children with Transcatheter and Surgically Closed Secundum Atrial Septal Defects (ASDs): A Strain Echocardiography Study

**DOI:** 10.3390/diagnostics15050606

**Published:** 2025-03-02

**Authors:** Serra Karaca, Doruk Özbingöl, Pelin Karaca Özer, Mustafa Lütfi Yavuz, Türkan Tansel, Kemal Nişli

**Affiliations:** 1 Pediatric Cardiology Department, Istanbul Faculty of Medicine, Istanbul 34093, Turkey; dorukozbingol@gmail.com (D.Ö.);; 2 Cardiology Department, Istanbul Faculty of Medicine, Istanbul 34093, Turkey; pkaracaozer@gmail.com (P.K.Ö.); mustafayavuz135@gmail.com (M.L.Y.); 3 Cardiovascular Surgery Department, Istanbul Faculty of Medicine, Istanbul 34093, Turkey; ttansel@istanbul.edu.tr

**Keywords:** atrial septal defect, strain echocardiography, electrocardiogram, right ventricle

## Abstract

**Background/Objectives**: Secundum-type atrial septal defect (ASD) is one of the most common congenital heart defects, with an incidence of 5.64 per 10,000 live births worldwide. In our study, long-term follow-up results of children who underwent percutaneous ASD closure and patients who underwent surgical treatment were evaluated using right ventricular strain echocardiography and electrocardiography. **Methods**: 30 patients who underwent transcatheter ASD closure and 30 patients provided with surgical ASD closure were prospectively compared with 50 healthy children with similar demographic characteristics. ECG and transthoracic echocardiography were performed for all patients. The evaluated echocardiography variables are Tricuspid annular plane systolic excursion (TAPSE), 2D right ventricle (RV) and right atrium (RA) dimensions, right ventricular segmental longitudinal strain, and global longitudinal strain. ECG evaluation was performed especially in terms of QRS duration and its correlation with strain echo measurements. **Results**: The surgical treatment group has statistically significant ASD size compared to patients who underwent transcatheter closure (20 ± 3.6 and 14.87 ± 3.7 mm, *p* < 0.001). Patients who had surgical treatment have increased RA and RV diameters, and a statistically significant decrease was observed in right ventricular free-wall longitudinal strain and right ventricular four-chamber longitudinal strain compared to patients in transcatheter and the control group (*p* < 0.001). QRS durations were similarly normal in electrocardiography in the transcatheter and the control groups, and the QRS duration was observed as statistically significantly increased in the patients in the surgical treatment group (*p* < 0.001). **Conclusions**: Strain values of the patients who underwent surgical closure were lower, and the QRS values on the ECG were longer, compared to the transcatheter group, which is an indicator that a large ASD diameter has a negative effect on long-term right ventricular function. With this in mind, we argue that early surgical closure is an appropriate treatment option for children whose ASD is large for their age and who are not suitable candidates for transcatheter treatment.

## 1. Introduction

Atrial septal defect (ASD) is one of the most common (10–15%) types of congenital heart defect in children [1]. The secundum-type ASD is one of the most common congenital heart defects, with an incidence of 5.64 per 10,000 live births worldwide. The female–male ratio for secundum ASDs is 2:1 [2]. There are essentially four types of ASDs, namely, ostium primum, ostium secundum, sinus venosus and coronary sinus.

Patients with ASD are often asymptomatic in childhood, but they may become symptomatic later in life. They have a left-to-right shunt, which causes chronic volume overload in the right atrium, right ventricle and pulmonary artery, leading to heart failure and pulmonary hypertension in adulthood. Atrial septum closure is recommended in patients with significant right heart volume overload, a shunt ratio > 1.5:1 or right atrium or right ventricle dilatation on an echocardiographic study. Percutaneous ASD closure has become the approach of choice for most secundum-type ASDs among interventional cardiologists all over the world, provided the defect is deemed suitable for the approach [3].

Transcatheter device closure of ASD is the preferred way to close septum secundum defects [4]. This is a safe technique, with low incidences of morbidity and mortality. The first transvenous device used to close an ASD was employed by King and Mills in 1974 [5], and, in the past 50 years, important interventions and innovations have occurred in the field of percutaneous ASD closure using transcatheter-based devices.

The closure of this defect causes a sudden change in a patient’s loading conditions in both ventricles. Compared to surgical closure, transcatheter ASD closure is associated with fewer complications and faster hemodynamic improvement. Decreases in right ventricular volume overload, right ventricular dimensions and pulmonary artery pressure (PAP) after transcatheter ASD closure result in prominent symptomatic improvement [6].

In our study, long-term follow-up results of children who underwent percutaneous ASD closure and patients who underwent surgical treatment were evaluated using right ventricular strain echocardiography and electrocardiography.

## 2. Materials and Methods

This prospective cross-sectional study evaluated the long-term follow-up transthoracic echocardiographic (TTE) results of ASD patients who underwent transcatheter and surgical closure in the Department of Pediatric Cardiology at Istanbul University between 2009 and 2020. Children were evaluated in our clinic between 2023 and 2024.

In this study, 30 patients who underwent transcatheter ASD closure and 30 patients provided with surgical ASD closure were prospectively compared with 50 healthy children with similar demographic characteristics. No other cardiac disease or cardiovascular risk factors were observed in either intervention group or in the control group. Patients without a residual shunt, arrhythmia and/or complications were included in the study. Patients with complex cardiac anomalies, cardiomyopathies, systemic diseases with cardiac involvement, multiple fenestrated atrial septal defects and aneurysmal septal tissue, or patients with missing data in their files, were excluded from the study.

The following devices were used for the procedures: Amplatzer^®^ Septal Occluder (AGA Medical Corp., Golden Valley, MN, USA), Occlutech^®^ Occluder (Occlutech GmbH, Jena, Germany), Solysafe^®^ Septal Occluder (Swissimplant AG, Solothurn, Switzerland), Cera^®^ Occluder (Lifetech Scientific Shenzen Co., Ltd., Shenzen, China) and Biostar^®^ Septal Occluder (NMT Medical, Boston, MA, USA).

The balloon-sizing measurement value was deemed the primary value in patients without a loose septum; however, patient-specific device selections were made considering the relationship between the total septum diameter, the type of defect and the patient’s age.

In our clinic, patients who undergo transcatheter and surgical ASD closures are evaluated with TTE at one month and six months after the procedure and then annually thereafter. In the present study, we selected patients who were followed up after a diagnosis of transcatheter or surgically closed ASD in our outpatient clinic, who continued to attend their routine check-ups and who had the procedure performed at least 3 years ago. We imposed these criteria because we aimed to evaluate the medium- to long-term results after the closure process and because patient groups were required to have similar follow-up periods. Since there were no baseline strain echocardiography values to use, a control group consisting of children of the same ages and genders was created to make an accurate comparison.

**Figure 1:**
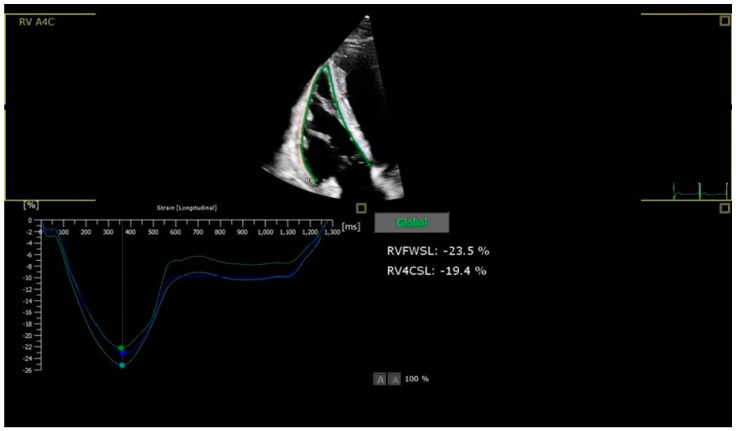
Strain echocardiography RV-FWSL and RV-4CSL images.

ECG readings taken at Istanbul University’s Faculty of Medicine were recorded with a Schiller electrocardiography device at a sampling rate of 1000 Hertz per second. A MEANS baseline correction was performed for each lead. The following ECG variables were evaluated: heart rate; P-wave amplitude in lead II; P-wave duration; PQ duration; QRS duration; and QT interval, corrected with the Bazett formula (QTc). A Philips Affiniti 70-3 echo machine equipped with a multifrequency 2.5–3.5 MHz. transthoracic echocardiography (TTE) was performed on all patients, and different modalities, such as 2D TTE and Tissue Doppler Imaging (TDI), were used. TAPSE and 2D RV and RA dimensions were measured to evaluate right ventricular functions. Right ventricular speckle-tracking strain echocardiography was performed to evaluate subclinical myocardial function. Right ventricular segmental longitudinal strain (SLS) and global longitudinal strain (GLS) measurements were made on the apical 4-chamber images taken (Figure 1). Strain measurements were evaluated with a computer program (TOMTEC Imaging Systems, Munich, Germany) corresponding with ethical approval code.

The Kolmogorov–Smirnov test was used to analyze the normality of the data. Parametric continuous data were expressed as mean ± standard deviation (SD), and non-parametric continuous data, median (minimum–maximum) and categorical data were expressed as percentages. A chi-square test was used to assess the differences in categorical variables among the groups. ANOVA analyses were performed to compare all reported data for parametric variables, whereas the Kruskal–Wallis test was used for comparisons among non-parametric variables among groups. The relationships among the parameters were assessed using Pearson’s or Spearman’s correlation analysis, according to the normality of the data. Logistic regression analysis was used to determine independent predictors for RV-FWLS and RV-4CLS. Significance was assumed with a two-sided value of *p* < 0.05. All statistical tests were conducted using the Statistical Package for the Social Sciences 26.0 for Windows (SPSS Inc., Chicago, IL, USA).

## 3. Results

The study included 30 patients who underwent ASD closure with the transcatheter method, dubbed Group 1, and 30 patients who underwent surgical closure, Group 2. The control group consisted of 50 healthy children with similar demographic characteristics (Table 1).

In Group 1, 19 (63%) of the patients were female; 11 (37%) were male. The average weights and heights of the patients were 55.8 ± 12.5 kg and 160.8 ± 10.6 cm; their average body mass index was 21.3 ± 2.5 kg. The mean age at the time of the procedure was 15.2 ± 1.8 years. In the echocardiographic measurements, their ASD diameter was found to be 14.87 ± 3.7 mm (min: 100, max: 22). ASD closure was performed transcatheterally using a 16.6 ± 3.4 mm device. The patients’ mean follow-up period was 8.03 ± 2 years (min: 4, max: 12).

In Group 2, 14 (47%) of the patients were male; 16 (53%) were female. The average weights and heights of the patients were 55.7 ± 13.6 kg and 159.4 ± 11.8 cm. Their average body mass index was 21.7 ± 3. In the echocardiographic measurements, their ASD diameter was 20 ± 3.6 mm (min: 13, max: 26). The mean age of the patients at the time of the procedure was 15.1 ± 1.9 years. The patients’ mean follow-up period was 8.38 ± 3.2 years (min: 3, max: 15).

The relationship between RV-FWLS, RV-4CLS and ASD size, echocardiographic parameters and QRS duration were evaluated via Spearman’s or Pearson’s correlation analyses (Table 2). A statistically significant correlation was detected between ASD size and both RV-FWLS and RV-4CLS (r = 0.771, *p* < 0.001; r = 0.621, *p* < 0.001, respectively). Again, QRS duration significantly correlated with RV-FWLS and RV-4CLS (r = 0.473, *p* < 0.001; r = 0.453, *p* < 0.001, respectively).

The parameters affecting impaired RV-FWLS and RV-4CLS (>−18) were evaluated by using logistic regression analysis with univariate and multivariate analysis (Table 3A,B). ASD size and QRS duration were thus determined to be independent predictors of impaired RV-FWLS (OR = 2.6, *p* < 0.001; OR = 1.1, *p* = 0.009, respectively) and RV-4CLS (OR = 1.5, *p* < 0.001; OR = 1.1, *p* = 0.008, respectively).

## 4. Discussion

Transcatheter closure in the treatment of ASD is preferred in secundum types because the risks associated with the procedure are lower, compared to surgery, and typical hospital stays are shorter. Surgical treatment should be preferred in cases of especially large defects; defects other than the secundum type; and in patients with multiple rim deficiencies, floppy septums, those with small left atria and those whose defect diameter is incompatible with the total septum because the risk of complications is high [7,8].

Studies comparing transcatheter and surgical treatment of secundum ASD have shown similar effectiveness. In a multicenter, nonrandomized study, transcatheter treatment was shown to be associated with fewer complications and shorter hospital stays compared to surgery [9]. A meta-analytical study of 13 prior works also showed a lower complication rate in transcatheter treatment [8].

The risks of transcatheter closure were reported in a meta-analysis that evaluated 203 studies involving 28,142 patients. Major complications for ASD were noted as device embolization and pericardial tamponade (1.6%). Minor complications included arrhythmias, such as transient heart blocks, vascular injuries, embolisms and similar vascular complications (1.6%). During follow-up, cerebrovascular events (1.3%) and device thrombosis (1.2%) were noted as the most common problems [10].

Long-term results after percutaneous ASD closure were evaluated in a multicenter study with 1326 pediatric patients, and, according to this study, the periprocedural complication rate was found to be 1.8%, and the late complication rate was 1%. Mortality and erosion have never been detected. The average follow-up period is 3.5 years (0.6–18 years). In total, 173 patients were followed for more than 10 years [11].

A body weight of less than 15 kg and ASD greater than 20 mm are predictors for the development of complications during percutaneous ASD closure. The long-term results after percutaneous ASD closure were evaluated in a multicenter study of 1326 pediatric patients, and the periprocedural complication rate was found to be 1.8%, while the late complication rate was 1%. Neither mortality nor erosion was detected. The average follow-up period in the study was 3.5 years (0.6–18 years), and 173 patients were followed for more than 10 years [12].

Changes in right ventricular geometry after transcatheter ASD closure often occur as part of the healing process. During this process, the size and shape of the right ventricle improve over time. However, the timeframe for recovery varies from patient to patient. After transcatheter ASD closure, the size of the right ventricle usually begins to improve within the first 6–12 months. Changes in the geometry of the right ventricle are accompanied by the disappearance of abnormal blood flow through the heart and a decrease in the load on the ventricle. During this process, a significant improvement in right ventricular wall thickness and dimensions can be observed. The healing process may take longer, especially for people with extensive ASDs or additional heart disease. It is important to monitor heart function and geometry regularly [13]. Echocardiographic evaluation results of surgical and transcatheter ASD closure methods in the medium- and long-term are important for determining and monitoring the closure method.

Strain echocardiography is an effective echocardiographic method used to evaluate the deformation and contractility performance of the heart muscle. When evaluating the results of both surgical and transcatheter ASD closure methods, strain echo can be used to evaluate in detail the medium- and long-term effects of both methods. Strain echo is an important tool for monitoring changes in the contractility and geometry of the right ventricle and allows patients to monitor their recovery process in detail. Long-term follow-ups are also important to ensure that right ventricular functions and geometry remain healthy [14,15]. An adult study by Dhillon et al. evaluated the effects of transcatheter and surgical ASD closure on right ventricular function. Cross-sectionally guided M mode echocardiographic ventricular long-axis function was measured prospectively before and within one week after ASD closure by device in 17 patients and by surgery in 12 patients and compared with 18 normal subjects. In this study, they found out that both systolic and diastolic right ventricular function was impaired due to cardiopulmonary bypass but was preserved after being closed with device [16]. Although our study had long-term results, similar to this study, the strain values of children who underwent surgical closure were found to be significantly impaired compared to both the transcatheter and control groups, and the QRS durations were longer on the ECG.

Bussadori et al. examined the effects of ASD closure on right ventricular geometry in long-term studies. They found that the geometry and functions of the right ventricle improve after both transcatheter and surgical closure. They compared right and left ventricular strain values before and after percutaneous ASD closure. Patients in the study group were evaluated the day before and 24 h after ASD closure. They noted a rapid decrease in right ventricular volumes and an increase in RV EF after the procedure. Right ventricular volumes decreased significantly in the first three days, and this change was positively correlated with improvement in both the LV ejection fraction and LV end-diastolic diameter. The improvement in RV volume continued for up to six months [17]. In our study, in contrast to Bussadori et al.’s findings, strain, RV volume, RV longitudinal and global strain values were measured approximately eight years after the procedure. Since our study’s patients did not have baseline strain values, the strain values of children who underwent transcatheter and surgical closure were compared with the strain values of healthy children of the same age.

In a study conducted in 2006, Eyskens et al. evaluated the longitudinal peak systolic velocities, peak systolic strain rate and end-systolic strain of the right ventricular free wall and middle segment of the septum before and after ASD device closure in 21 pediatric patients and compared them with a control group. In that work, RV deformation indices in the patient group were no different from the control group and did not change after ASD device closure. They concluded that strain and strain rate values were independent of volume load, while myocardial velocities were load-dependent because these velocities were clearly reduced after the ASD device closure compared to the control group. They even suggested that the device inside the atrial septum may affect interventricular septum deformation and reduce the function of the septum [18].

In our study, we compared long-term global longitudinal strain and right ventricular free-wall strain values by comparing ASD patients in two groups who underwent surgical and percutaneous device closure and with a control group. We evaluated right ventricular functions by examining the strain echocardiographic and electrocardiographic findings of patients in a similar age group who underwent surgical and transcatheter ASD closure.

Moradian et al. [19] found that, after transcatheter ASD closure, strain echo measurements of the right atrium and right ventricle showed a significant improvement compared to patients who underwent surgical closure. It appears that the strain and strain rate parameters on the right ventricle had statistically similar findings to the control group.

In a study conducted on adult patients, Alkhateeb et al. [20] evaluated the effect of the device diameter used in atrial septal defect closure on biventricular function using strain echocardiography. Their patients were divided into two groups, with average device diameters of 16.1 mm and 29.5 mm. They found a negative correlation between a large device diameter and improvement in RV global longitudinal strain values in the early period. In our study, the defect diameter of the patients who underwent transcatheter closure was 14.87 ± 3.7 mm, and the average device diameter was 16.6 ± 3.4 mm. In our surgical closure group, the defect diameter was 20 ± 3.6 mm. The fact that the strain values in the surgical group were significantly lower than the transcatheter group, when comparing the two groups, suggested that the baseline ASD diameter and post-closure strain values showed a negative correlation; that is, the ASD diameter is an important determinant in the long-term follow-up of right ventricular functions after closure.

After successful percutaneous atrial septal defect closure, changes in heart rate, P-wave amplitude, PQ duration and QRS duration were observed in both children and adults in a study by Kamphuis et al. [21]. A decrease in the QTc interval was also observed. Although QRS duration and QTc interval changes occur later, other changes were shown to occur immediately after closure. In our study, we observed that the QRS duration in patients who underwent surgical ASD closure was statistically significantly longer than in patients who underwent transcatheter ASD closure and the control group.

Studies by Grignani et al. [22] and Thilén et al. [23] found right-sided volume overload due to atrial left-to-right shunting causes changes in the 12-lead ECG due to mechano-electrical coupling, leading to both atrial and ventricular strain. RA dilatation can cause increased P-wave amplitude, prolonged P-wave duration and increased P-wave dispersion due to delayed atrial conduction; all of these are useful markers for predicting atrial arrhythmias. RV dilatation can cause prolonged QRS duration, right bundle branch block and hookage (a notch near the top of the R-wave in the lower extremity leads). Recently, it has been shown that RV volume overload can cause a prolongation of the QTc interval in patients with ASD [24]. After successful ASD closure, right-sided volume overload has been shown to resolve within 24 h, initiating the geometric remodeling of the right atrium and ventricle. This geometric remodeling should continue for up to 6–8 weeks after percutaneous ASD closure. A direct decrease in heart rate has been shown after ASD closure, and this decrease persists in the long term [25].

In a study conducted by Kamphuis et al., ECG after percutaneous ASD closure in children and adults showed a decrease in heart rate, P-wave amplitude, PQ duration, QRS duration and QTc interval. Except for late QRS duration and QTc interval changes, most changes occurred directly after device closure. The spatial QRS-T angle decreased significantly in children at late follow-up. After a successful percutaneous atrial septal defect closure, children showed a decrease in heart rate, P-wave amplitude, PQ duration, QRS duration and QTc interval. The changes mostly occurred immediately after ASD closure, but QRS duration and QTc interval changes occurred after a longer period [21].

It should be noted that variability among vendor software in strain echocardiography, along with operator experience, may influence the findings [26,27]. However, the generalizability of our results may be limited due to the single-center design and relatively small sample size. Future multicenter studies with larger cohorts are necessary to validate these findings and enhance their clinical relevance.

## 5. Conclusions

Both ASD resolution methods have the potential to improve right heart function, but long-term follow-up and evaluation are important, considering individual patients’ responses and their risk of complications. According to the results of our study, the fact that the strain values of the patients who underwent surgical closure were lower and the QRS values on the ECG were longer, compared to the transcatheter group, is an indicator that a large ASD diameter has a negative effect on long-term right ventricular function. With this in mind, we argue that early surgical closure is an appropriate treatment option for children whose ASD is large for their age and who are not suitable candidates for transcatheter treatment.

## Figures and Tables

**Table 1:** Statistical analysis of patients.

Variable (n)	Control (50)	Transcatheter (30)	Surgical (30)	*p*-Value
Age	14.8 ± 2.1	15.2 ± 1.8	15.1 ± 1.9	0.62
Male sex, n (%)	22 (44%)	11 (37%)	16 (53%)	0.43
Body mass index (BMI)	21.3 ± 2.5	21.3 ± 2.5	21.7 ± 3.1	0.8
Atrial septal defect (ASD) size (mm)	---	14.87 ± 3.7	20 ± 3.6	<0.001
Right ventricular free-wall longitudinal strain (RV-FWLS)	−23.6 ± 3.3 ^xy^	−20.3 ± 2 ^xz^	−16.90 ± 6.6 ^yz^	<0.001
Right ventricular four-chamber longitudinal strain (RV-4CLS)	−23.3 ± 2.9 ^xy^	−20.5 ± 2.8 ^xz^	−17.1 ± 2.1 ^yz^	<0.001
Tricuspid annular plane systolic excursion (TAPSE)	17.9 ± 2 ^xy^	19.6 ± 2.5 ^xz^	14.5 ± 1.9 ^yz^	<0.001
Systolic pulmonary artery pressure (sPAP) (mmHg)	16 ± 1	16.1 ± 1	16.3 ± 1.6	0.96
Right ventricle (mm)	34.6 ± 2 ^y^	35.5 ± 1.8	36.5 ± 2.1 ^y^	0.001
Right atrium (mm)	31.8 ± 2.4 ^xy^	32.6 ± 2 ^xz^	34.1 ± 2 ^yz^	<0.001
Left atrium (mm)	33.8 ± 2.4	34.9 ± 1.6	34.6 ± 1.6	0.054
Left ventricle end diastolic diameter (LVEDD) (mm)	46.4 ± 3.8	47.3 ± 4.6	47.6 ± 3.7	0.41
Interventricular septum (IVS) (mm)	8.5 ± 0.8	8.75 ± 0.8	8.53 ± 0.9	0.38
Left ventricle ejection fraction (LVEF)	67.6 ± 3.7	66.8 ± 3.8	68.1 ± 3.1	0.39
QRS (msec)	74.4 ± 12 ^y^	77.7 ± 17.6 ^z^	96.3 ± 17.7 ^yz^	<0.001
Device size (mm)	---	16.6 ± 3.4	---	---
Sizing balloon (mm)	---	16.1 ± 3.3	15.1	0.62
Right bundle branch block (RBBB), n (%)	0	1 (3%)	3 (10%)	0.07
Follow-up period		8.03 ± 2	8.38 ± 3.2	0.6

^x^ Statistically significant difference between control group and transcatheter group; ^y^ statistically significant difference between control group and surgical group; ^z^ statistically significant difference between transcatheter group and surgical group.

**Table 2:** Right ventricular strain and echocardiographic variable correlations.

Correlation Analysis				
Variables	RV-FWLS		RV-4CLS	
	r	*p* Value	r	*p* Value
RV-FWLS	1	---	0.883	<0.001
RV-4CSL	0.883	<0.001	1	---
Age	−0.131	0.17	−0.088	0.36
BMI	−0.091	0.35	0.002	0.98
ASD size	0.771	<0.001	0.621	<0.001
TAPSE	−0.572	<0.001	−0.533	<0.001
sPAP	0.239	0.012	0.228	0.016
RV	0.197	0.039	0.254	0.007
RA	0.241	0.011	0.244	0.01
LA	0.033	0.73	0.014	0.89
LVEDD	−0.014	0.88	0.083	0.39
IVS	−0.139	0.15	−0.064	0.51
LVEF	0.11	0.27	0.11	0.25
QRS (msec)	0.473	<0.001	0.453	<0.001

**Table 3:** (**A**). Univariate logistic regression analysis of predictors of right ventricular strain after atrial septal defect closure. (**B**). Multivariate logistic regression analysis of predictors of right ventricular strain after atrial septal defect closure.

**(A)**						
**Variables**	**Univariate Logistic Regression**					
	**RV-FWLS**			**RV-4CLS**		
	**OR**	**95% CI**	***p* Value**	**OR**	**95% CI**	***p* Value**
Age	1.085	0.84–1.4	0.53	1.019	0.81–1.28	0.87
BMI	1.145	0.95–1.4	0.15	1.158	0.98–1.37	0.09
ASD Size	2.073	1.41–3.1	<0.001	1.545	1.25–1.91	<0.001
TAPSE	0.460	0.32–0.67	<0.001	0.462	0.32–0.66	<0.001
sPAP	1.717	1.15–2.6	0.009	1.458	1.01–2.11	0.045
RV	1.818	1.28–2.58	0.001	1.798	1.3–2.49	<0.001
RA	1.636	1.22–2.2	0.001	1.533	1.18–1.98	0.001
LA	1.177	0.91–1.5	0.21	1.185	0.94–1.5	0.16
LVEDD	1.107	0.98–1.25	0.01	1.131	1.01–1.27	0.034
IVS	1.051	0.58–1.89	0.87	1.257	0.74–2.14	0.4
LVEF	1.012	0.88–1.2	0.87	1.079	0.95–1.23	0.25
RBBB	16.875	1.65–172.5	0.017	12.143	1.2–122.7	0.034
QRS (MS)	1.179	1.1–1.27	<0.001	1.203	1.11–1.3	<0.001
Device Type	1.856	0.29–11.8	0.51	1.461	0.53–4	0.46
**(B)**						
**Variables**	**Multivariate Logistic Regression**					
	**RV-FWLS**			**RV-4CLS**		
	**OR**	**95% CI**	***p* Value**	**OR**	**95% CI**	***p* Value**
ASD Size	2.6	1.3–5.2	<0.001	1.5	1.09–1.9	<0.001
QRS (msec)	1.1	1.04–1.3	0.009	1.1	1.03–1.2	0.008

## Data Availability

The raw data supporting the conclusions of this article will be made available by the authors on request.

## Abbreviations

The following abbreviations are used in this manuscript:

ASD	Atrial septal defect
TTE	Transthoracic echocardiography
TDI	Tissue Doppler Imaging
RV-SLS	Right ventricular segmental longitudinal strain
GLS	Global longitudinal strain
TAPSE	Tricuspid annular plane systolic excursion
RV-FWLS	Right ventricular free-wall longitudinal strain
RV-4CLS	Right ventricular four-chamber longitudinal strain

## References

[1] Van der Linde D., Konings E.E., Slager M.A., Witsenburg M., Helbing W.A., Takkenberg J.J., Roos-Hesselink J.W. (2011). Birth prevalence of congenital heart disease worldwide: A systematic review and meta-analysis. J. Am. Coll. Cardiol..

[2] Hoffman J.I., Kaplan S. (2002). The incidence of congenital heart disease. J. Am. Coll. Cardiol..

[3] Silvestry F.E., Cohen M.S., Armsby L.B., Burkule N.J., Fleishman C.E., Hijazi Z.M., Lang R.M., Rome J.J., Wang Y. (2015). Guidelines for the Echocardiographic Assessment of Atrial Septal Defect and Patent Foramen Ovale: From the American Society of Echocardiography and Society for Cardiac Angiography and Interventions. J. Am. Soc. Echocardiogr..

[4] Butera G., Carminati M., Chessa M., Youssef R., Drago M., Giamberti A., Pomè G., Bossone E., Frigiola A. (2006). Percutaneous versus surgical closure of secundum atrial septal defect: Comparison of early results and complications. Am. Heart J..

[5] King T.D., Thompson S.L., Steiner C., Mills N.L. (1976). Secundum atrial septal defect. Nonoperative closure during cardiac catheterization. JAMA.

[6] Ghaderian M., Sabri M.R., Ahmadi A.R., Alipour M.R., Dehghan B., Mehrpour M. (2019). Midterm Follow-up Results of Transcatheter Interatrial Septal Defect Closure. Heart Views.

[7] Ko H.K., Kang S.Y., Yu J.J., Ko J.K., Kim Y.H. (2015). Small left atrial size complicating percutaneous transcatheter device closure of secundum atrial septal defect with conventional approach. Korean Circ. J..

[8] Butera G., Biondi-Zoccai G., Sangiorgi G., Abella R., Giamberti A., Bussadori C., Sheiban I., Saliba Z., Santoro T., Pelissero G. (2011). Percutaneous versus surgical closure of secundum atrial septal defects: A systematic review and meta-analysis of currently available clinical evidence. EuroIntervention.

[9] Du Z.D., Hijazi Z.M., Kleinman C.S., Silverman N.H., Larntz K. (2002). Comparison between transcatheter and surgical closure of secundum atrial septal defect in children and adults: Results of a multicenter nonrandomized trial. J. Am. Coll. Cardiol..

[10] Abaci A., Unlu S., Alsancak Y., Kaya U., Sezenoz B. (2013). Short and long term complications of device closure of atrial septal defect and patent foramen ovale: Meta-analysis of 28,142 patients from 203 studies. Catheter. Cardiovasc. Interv..

[11] Fraisse A., Latchman M., Sharma S.R., Bayburt S., Amedro P., di Salvo G., Baruteau A.E. (2018). Atrial septal defect closure: Indications and contra-indications. J. Thorac. Dis..

[12] Jalal Z., Hascoët S., Gronier C., Godart F., Mauri L., Dauphin C., Lefort B., Lachaud M., Piot D., Dinet M.L. (2018). Long-Term Outcomes After Percutaneous Closure of Ostium Secundum Atrial Septal Defect in the Young: A Nationwide Cohort Study. JACC Cardiovasc. Interv..

[13] Balcı K.G., Balcı M.M., Aksoy M.M., Yılmaz S., Aytürk M., Doğan M., Yeter E., Akdemir R. (2015). Remodeling process in right and left ventricle after percutaneous atrial septal defect closure in adult patients. Turk. Kardiyol. Dern. Ars..

[14] Di Salvo G., Pacileo G., Castaldi B., Gala S., Morelli C., D’Andrea A., Limongelli G., Del Gaizo F., Merlino E., Russo M.G. (2008). Two-dimensional strain and atrial function: A study on patients after percutaneous closure of atrial septal defect. Eur. J. Echocardiogr..

[15] Jategaonkar S.R., Scholtz W., Butz T., Bogunovic N., Faber L., Horstkotte D. (2009). Two-dimensional strain and strain rate imaging of the right ventricle in adult patients before and after percutaneous closure of atrial septal defects. Eur. J. Echocardiogr..

[16] Dhillon R., Josen M., Henein M., Redington A. (2002). Transcatheter closure of atrial septal defect preserves right ventricular function. Heart.

[17] Bussadori C., Oliveira P., Arcidiacono C., Saracino A., Nicolosi E., Negura D., Piazza L., Micheletti A., Chessa M., Butera G. (2011). Right and left ventricular strain and strain rate in young adults before and after percutaneous atrial septal defect closure. Echocardiography.

[18] Eyskens B., Ganame J., Claus P., Boshoff D., Gewillig M., Mertens L. (2006). Ultrasonic strain rate and strain imaging of the right ventricle in children before and after percutaneous closure of an atrial septal defect. J. Am. Soc. Echocardiogr..

[19] Moradian M., Daneshamooz H., Shojaeifard M., Ghadrdoost B., Langeroudi H.M., Khorgami M.R. (2018). Echocardiographic Right Ventricular Deformation Indices Before and After Atrial Septal Defect Closure: A Scomparison between Device and Surgical Closure. Res. Cardiovasc. Med..

[20] Alkhateeb A., Roushdy A., Hasan-Ali H., Kishk Y.T., El Sayegh A., Hassan A.K.M. (2022). Impact of atrial septal defect device size on biventricular global and regional function: A two-dimensional strain echocardiographic study. Cardiol. Young.

[21] Kamphuis V.P., Nassif M., Man S.C., Swenne C.A., Kors J.A., Vink A.S., Ten Harkel A.D.J., Maan A.C., Mulder B.J.M., de Winter R.J. (2019). Electrical remodeling after percutaneous atrial septal defect closure in pediatric and adult patients. Int. J. Cardiol..

[22] Grignani R.T., Tolentino K.M., Rajgor D.D., Quek S.C. (2015). Longitudinal evaluation of P-wave dispersion and P-wave maximum in children after transcatheter device closure of secundum atrial septal defect. Pediatr. Cardiol..

[23] Thilén U., Carlson J., Platonov P.G., Olsson S.B. (2009). Atrial myocardial pathoelectrophysiology in adults with a secundum atrial septal defect is unaffected by closure of the defect. A study using high resolution signal-averaged orthogonal P-wave technique. Int. J. Cardiol..

[24] Rücklová K., Koubský K., Tomek V., Kubuš P., Janoušek J. (2016). Prolonged repolarization in atrial septal defect: An example of mechanoelectrical feedback due to right ventricular volume overload. Heart Rhythm..

[25] Monfredi O., Luckie M., Mirjafari H., Willard T., Buckley H., Griffiths L., Clarke B., Mahadevan V.S. (2013). Percutaneous device closure of atrial septal defect results in very early and sustained changes of right and left heart function. Int. J. Cardiol..

[26] Mirea O., Pagourelias E.D., Duchenne J., Bogaert J., Thomas J.D., Badano L.P., Voigt J.U. (2018). Intervendor Differences in the Accuracy of Detecting Regional Functional Abnormalities: A Report From the EACVI-ASE Strain Standardization Task Force. JACC Cardiovasc. Imaging.

[27] Negishi T., Negishi K., Thavendiranathan P., Cho G.Y., Popescu B.A., Vinereanu D., Kurosawa K., Penicka M., Marwick T.H. (2017). Effect of Experience and Training on the Concordance and Precision of Strain Measurements. JACC Cardiovasc. Imaging.

